# Efficacy of *Moringa oleifera* leaf powder as a hand- washing product: a crossover controlled study among healthy volunteers

**DOI:** 10.1186/1472-6882-14-57

**Published:** 2014-02-14

**Authors:** Belen Torondel, David Opare, Bjorn Brandberg, Emma Cobb, Sandy Cairncross

**Affiliations:** 1 Department of Disease Control, Faculty of Infectious and Tropical Diseases, London School of Hygiene and Tropical Medicine, Keppel Street, London WC1E 7HT, UK; 2 SBI Consulting Lda, Av V Lenine 3092-1E, Maputo, Mozambique

**Keywords:** *Moringa oleifera*, *Hand-washing product*, *Antibacterial*, *Diarrhoea*, *Tropical countries*

## Abstract

**Background:**

*Moringa oleifera* is a plant found in many tropical and subtropical countries. Many different uses and properties have been attributed to this plant, mainly as a nutritional supplement and as a water purifier. Its antibacterial activity against different pathogens has been described in different *in vitro* settings. However the potential effect of this plant leaf as a hand washing product has never been studied. The aim of this study is to test the efficacy of this product using an *in vivo* design with healthy volunteers.

**Methods:**

The hands of fifteen volunteers were artificially contaminated with *Escherichia coli. Moringa oleifera* leaf powder was tested as a hand washing product and was compared with reference non-medicated liquid soap using a cross over design following an adaptation of the European Committee for Standardization protocol (EN 1499). In a second part of tests, the efficacy of the established amount of *Moringa oleifera* leaf powder was compared with an inert powder using the same protocol.

**Results:**

Application of 2 and 3 g of dried *Moringa oleifera* leaf powder (mean log_10_-reduction: 2.44 ± 0.41 and 2.58 ± 0.34, respectively) was significantly less effective than the reference soap (3.00 ± 0.27 and 2.99 ± 0.26, respectively; p < 0.001). Application of the same amounts of *Moringa oleifera* (2 and 3 g) but using a wet preparation, was also significantly less effective than reference soap (p < 0.003 and p < 0.02, respectively). However there was no significant difference when using 4 g of *Moringa oleifera* powder in dried or wet preparation (mean log_10_-reduction: 2.70 ± 0.27 and 2.91 ± 0.11, respectively) compared with reference soap (2.97 ± 0.28). Application of calcium sulphate inert powder was significantly less effective than the 4 g of *Moringa oleifera* powder (p < 0.01).

**Conclusion:**

Four grams of *Moringa oleifera* powder in dried and wet application had the same effect as non-medicated soap when used for hand washing. Efficacious and available hand washing products could be useful in developing countries in controlling pathogenic organisms that are transmitted through contaminated hands.

## Background

Diarrhoeal diseases are a common cause of morbidity and the leading cause of death among children under five, accounting for 19% of mortality in this age group [1]. Most of all diarrhoeal deaths of children under five years are in Africa and South East Asia [2]. The vast majority of diarrheal diseases are caused by bacteria, viruses and protozoa, mainly found in human faeces which are spread from the stool of one person to the mouth of another. Hands can act as a vector for transmission of faecal pathogens, either via direct person-to-person transmission or by contaminating food that is later consumed [3]. Hand washing after defecation and before handling food is therefore a biologically plausible mechanism for interrupting pathogen transmission. Hands are also transmission vectors for respiratory infections, and interventions promoting hand hygiene have been shown to reduce gastrointestinal and respiratory illnesses by an average of 31% and 21%, respectively [4].

**Figure 1:**
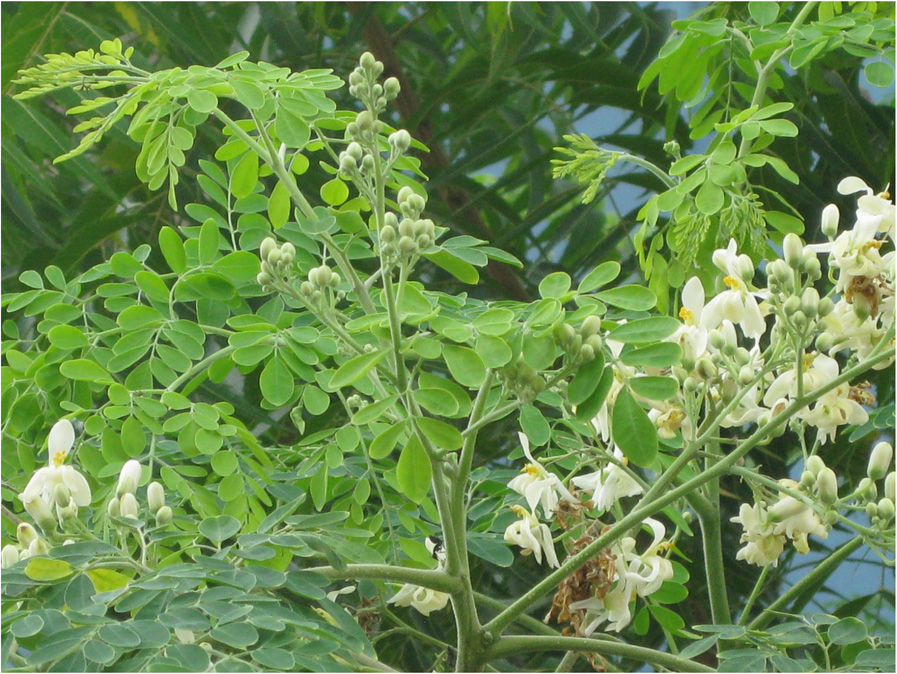
***Moringa oleifera *** **tree leaf.**

*Moringa oleifera* is a plant that can grow to 5–10 meters in a year and it is cultivated in many countries from South Asia to West and East Africa [5] (Figure 1). While it grows best in dry, sandy soil, it tolerates poor soil, including coastal areas. It is a fast-growing, drought-resistant tree that is native to the southern foothills of the Himalayas in northwestern India. Over the past two decades, many reports have appeared in mainstream scientific journals describing the nutritional and medicinal properties of *Moringa oleifera,* including treatment for malaria and intestinal worms, antifungal properties, malnutrition and also as a water purifier [6-10].

*Moringa oleifera* has been described to have antibacterial activity against some human bacterial pathogens, Gram-negative: *Shigella shinga*, *Pseudomonas aeruginosa*, *Shigella sonnei*, and *Pseudomonas spp*. and also Gram-positive: *Staphylococcus aureus*, *Bacillus cereus*, *Streptococcus B-haemolytica*, *Bacillus subtilis, Sarcina lutea* and *Bacillus megaterium*[11]. This effect has been attributed to different parts of the plant, such as the leaves, flowers, seeds, roots, fruit peel and unripe pods [5].

There is anecdotal evidence supporting the effect of this plant as an antibacterial agent and its use as a hand washing product [11-13]. However, there are no *in vivo* studies demonstrating the efficacy of *Moringa oleifera* leaf powder as a hand-washing product. The aim of this study is to test the efficacy of this product *in vivo* with healthy volunteers under laboratory controlled conditions.

## Methods

### Micro-organism

In order to test the efficacy of *Moringa oleifera* leaf powder, we used an adapted protocol of the European Committee for Standardization (EN 1499) [14] which is designed to evaluate the ability of hand-wash agents to eliminate transient pathogens form volunteers’ hands without regard to resident micro-organisms. This procedure is based on the “post-contamination treatment” of hands, and involves the placement of the test organism (*Escherichia coli (ACTC 25922)*) on the hands of test subjects followed by exposure to the test formulation. These methods are useful in testing the performance of products used in routine hand hygiene in health care centres.

### Subjects

The study was performed in the Medical Microbiology Laboratory of the London School of Hygiene and Tropical Medicine (LSHTM) from June to July 2012, and was approved by LSHTM Ethics Committee on 19th April 2012.

Fifteen adult volunteers from LSHTM were selected for the study, and a formal written consent was received from all of them. The volunteers were physically examined to ensure they were healthy with healthy skin. None had skin disorders like eczema, paronychia, scabies, abrasions, lacerations or skin allergy. They all had short fingernails with no artificial nails. They had no history of drug allergy and had not taken any systemic antibiotic in the two weeks prior to the study, which could otherwise impair the efficacy of the product being tested. All forms of jewellery were removed from their hands prior to hand washing, since it had the potential of retaining some bacteria, which could affect the recovery pre and post values.

### *Moringa oleifeira* preparations

*Moringa oleifera* leaf powder (100% natural) was obtained from LUTOR Ltd. (UK). The aim of this study was to test the efficacy of the leaves as a hand washing product in dried and wet preparation and also to test the effect of using different amounts (2, 3 and 4 g).

Because we wanted to test more than one preparation, we used a cross-over design with 5 groups of 3 subjects each. For day one, all the three volunteers in the first group used 5 ml of non–medicated liquid soap, 2 g of dry and wet *Moringa oleifera* powder as a hand washing product. The other four groups of volunteers repeated the same procedure on days 2, 3, 4 and 5.

During the second and third experiments (which were carried out the same day for each group), the aforementioned procedure was repeated with increasing amounts of *Moringa oleifera* powder; 3 and 4 g respectively. However, the quantity of non -medicated liquid soap was maintained at 5 ml. At the end of the whole series of runs every subject had used each hand-washing product once (Table 1).

**Table 1:** ***In vivo *** **study: cross-over arrangement of the *Moringa oleifera *** **treatments and non-medicated soap control**

		**Day 1**	**Day 2**	**Day 3**	**Day 4**	**Day 5**
Experiment 1 (n=15)	2 g wet Moringa	Group 1 (n=3)	Group 2 (n=3)	Group 3 (n=3)	Group 4 (n=3)	Group 5 (n=3)
	2 g dry Moringa					
	Soap*					
Experiment 2 (n=15)	3 g wet Moringa	Group 1 (n=3)	Group 2 (n=3)	Group 3 (n=3)	Group 4 (n=3)	Group 5 (n=3)
	3 g dry Moringa					
	Soap*					
Experiment 3 (n=15)	4 g wet Moringa	Group 1 (n=3)	Group 2 (n=3)	Group 3 (n=3)	Group 4 (n=3)	Group 5 (n=3)
	4 g dry Moringa					
	Soap*					

*non medicated soap (5 ml).

#### Contamination procedure

The hands of each volunteer were washed with a non-medicated soap, dried and immersed for 5 seconds in a contamination fluid which contained non-pathogenic *Escherichia coli (ACTC 25922)* 8.3×10^8^ cfu/ml. Excess of fluid was drained off and hands were air-dried for 3 min.

### Pre value

Bacteria were recovered for the initial pre value by kneading the fingertips of each hand separately for 60 seconds in 10 ml of tryptone soya broth (TSB) without neutralizers. The pre values were estimated using the Miles *et al*. technique [15].

### Hygienic hand washing procedure

The hands of two thirds of the volunteers were treated with the *Moringa oleifera* for one minute; the hands of the other third were washed with the reference solution (5 ml of non-medicated liquid soap) for one minute. Hands treated with dried *Moringa oleifera* received one of the 3 different amounts (2, 3 or 4 g) whilst hands treated with wet *Moringa oleifera* received 2, 3 or 4 g of the herb powder plus 20 ml of tap water which was added on the top of the *Moringa* powder deposited previously into each pair of hands. In both cases the participants then rubbed their hands with the product for 1 minute. Hands were rinsed under running water for 15 seconds and were allowed to dry for 3 minutes.

### Post values

After drying of the hands, the thumbs and fingers of both left and right hands were rubbed in separate petri dishes containing 10 ml of TSB for 60 seconds. Post values were determined using the method of Miles *et al*. [15]. After the procedure, the volunteers were given medicated soap to wash their hands before going home.

In order to test whether *Moringa oleifera* powder efficacy could be caused by mechanical friction with the hands, we compared the efficacy of 4 g of *Moringa oleifera* powder with an inert dried powder (calcium sulphate powder). The same protocol described before was used.

#### Statistical analysis

For both reference and test products, log counts from the left and right hands of each subject were averaged separately, for both pre and post values. The arithmetic means of all individual log_10_ reduction values were calculated. Statistical analysis was performed with the Statistical Package STATA version 11.0. First it was checked whether the data were normally distributed using Kurtosis and Skewness test. Since the data did not follow a normal distribution, the Wilcoxon matched-pair signed ranks test was used to test for differences between each *Moringa oleifera* preparation and the reference soap and also between *Moringa oleifera* and the inert powder. The new product (Moringa oleifera) was considered to have the same efficacy as the reference product (soap) if the mean log10 reduction factor was not significantly smaller for the former than for the latter. Because of the confirmative nature of the test on this application, the level of significance is set at p = 0.1. The test is to be used one-sided. The discrimination efficiency of the test procedure described has been set to detect a difference between the two mean log reduction factors of approximately 0.6 log at a power of 95%.

## Results

Application of 2 and 3 g of dried *Moringa oleifera* leaf powder resulted in a mean log_10_-reduction of 2.44 ± 0.41 and 2.58 ± 0.34, respectively. The reduction observed was significantly less effective than with the reference soap (3.00 ± 0.27 and 2.99 ± 0.26, respectively; p < 0.001) (Table 2). Application of the same amounts of *Moringa oleifera* (2 and 3 g) but using a wet preparation was also significantly less effective than reference soap (p < 0.003 and p < 0.02, respectively). However, when using 4 grams of *Moringa oleifera* powder in dried or wet preparation there was no significant difference (mean log_10_-reduction: 2.70 ± 0.27 and 2.91 ± 0.11, respectively) compared with reference soap (2.97 ± 0.28, p > 0.5).

**Table 2:** **Mean log reduction factor of *E. coli *** **after volunteers washing with *Moringa oleifera *** **product or reference product (non-medicated liquid soap)**

**Product**	**Mean log reduction factor of Moringa product (SD)**	**Mean log reduction factor of reference soap (SD)**	**Difference**	**p**
Moringa 2 g				
Dry	2.44 (0.41)	3.00 (0.27)	0.56	p=0.00001
Wet	2.70 (0.31)	3.00 (0.27)	0.30	p=0.003
Moringa 3 g				
Dry	2.58 (0.34)	2.99 (0.26)	0.41	p=0.0001
Wet	2.84 (0.32)	2.99 (0.26)	0.15	p=0.03
Moringa 4 g				
Dry	2.70 (0.27)	2.97 (0.28)	0.27*	p=0.06
Wet	2.91 (0.11)	2.97 (0.28)	0.07	p=0.16

P values were derived using Wilcoxon’s matched pairs signed-rank tests.

*significantly difference between dry and wet preparations (p=0.01).

**Figure 2:**
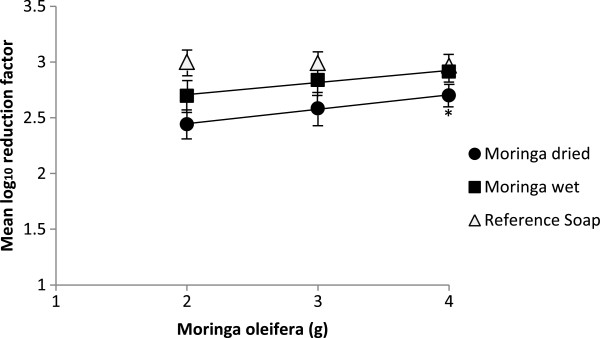
**Mean log**_**10 **_**reduction factor of *E. coli *after volunteers washed their hands with different doses of *Moringa oleifera *and using dried and wet preparations.** Amount of reference soap applied was always 5 ml.

When we compared the efficacy of 2, 3 and 4 g of dried vs. wet *Moringa oleifera*, our results showed that *Moringa oleifera* was more efficacious in reducing bacterial contamination when used in wet preparation in all the 3 doses, but the difference was significant (0.21 log_10_ higher for the wet than the dried preparation; p = 0.01) only when 4 g of *Moringa oleifera* was used (Table 2). We also observed higher effect when we increased the dose of *Moringa oleifera* in both wet and dried preparations (Figure 2). The mean log_10_-reduction with 4 g of dried *Moringa oleifera* was significantly greater than with 2 and 3 g dried preparation (p < 0.01). 4 g of wet *Moringa oleifera* was more efficacious than 2 and 3 g of wet preparation but the reduction was only significantly higher when compared with 2 g of wet *Moringa oleifera* (0.21 log_10_ reduction (p = 0.01).

We wanted to test whether the *Moringa oleifera* bacterial reduction effect was due to the mechanical friction of hands rubbed when using dried powder. Therefore we selected calcium sulphate as an inert and sterile powder and compared its efficacy with 4 g of *Moringa oleifera* in dried and wet preparation. The results are shown in Table 3. Application of 4 g of dried calcium sulphate resulted in a mean log_10_-reduction of 2.33 ± 0.35 less than those obtained with 4 g of dried and wet *Moringa oleifera* (2.70 ± 0.27 and 2.91 ± 0.11, respectively, p < 0.01). The reduction observed was also significantly less than with the reference soap; p < 0.001).

**Table 3:** **Mean log**_**10 **_**reduction factor of E. *coli *** **after volunteers washing with 4 g of *Moringa oleifera *** **product, reference product (non-medicated liquid soap) or inert powder (calcium sulphate)**

**Product**	**Mean log reduction factor of Moringa or ref soap (SD)**	**Mean log reduction factor of inert product (SD)**	**Difference**	**P**
Moringa 4 g dry	2.70 (0.27)	2.33 (0.35)	0.37	p=0.02
Moringa 4 g wet	2.91 (0.11)	2.33 (0.35)	0.58	p=0.0001
Reference soap	2.97 (0.28)	2.33 (0.35)	0.64	p=0.000002

## Discussion

The main finding of this study is that *Moringa oleifera* can reduce bacterial load on artificially contaminated hands when used as a dried or wet powder formulation. A dose of 4 g of *Moringa oleifera* in wet or dried formulation presented the same efficacy as non-medicated soap.

Different studies have shown an *in vitro* antibacterial effect from *Moringa oleifera* against different bacteria. Peixoto et al. [16] evaluated the antibacterial effect of aqueous and ethanolic *Moringa oleifera* leaf extracts on the growth of gram-positive and negative bacteria, and they found that some strains of *E. coli, P. aeruginosa* and *S. Enteritidis* were resistant to all the *Moringa oleifera* preparations but the same extracts were most effective against *S.aureus*, *V. parahaemolyticus*, *E. faecalis* and *A. caviae*. Other authors found that aqueous and ethanolic *Moringa oleifera* leaf extracts were effective against *Salmonella*[10]*.* The authors attributed the antibacterial effect to the presence of saponins, tannic, phenolic and alkaloid phytoconstituents [16]. *Moringa oleifera* leaves had been found in other studies to present an important amount of saponins (80 g/kg) [17]. Saponins are chemical compounds which have detergent or surfactant properties [18] and this may explain the bacterial reduction when using *Moringa oleifera* as a hand washing product.

In this study we also showed a dose–response reduction effect of *Moringa oleifera*, with a dose of 4 g associated with a greater bacterial reduction, comparable with the efficacy of non-medicated soap. These results suggest that when using *Moringa oleifera*, the hands have to be completely full of the powder in order to have the best results. The use of a wet preparation also presented better results than when dried powder was used. This could be explained by the water’s effect [19] in extracting more of the active component of the plant at the moment of hand cleansing. Water is a universal solvent, and other many studies have described its ability to extract plant products with antimicrobial activity [19]. Thus the solvents most commonly used for preliminary investigations of antimicrobial activity in plants are methanol, ethanol and water [20-22].

Different studies have found that mechanical friction applied during hand washing plays a role in removing microorganisms adhering to hands [23]. Therefore we tested whether the effect of *Moringa oleifera* leaf powder could be due only to the mechanical friction exerted when rubbing the hands together. For this purpose we compared the effect of 4 g of *Moringa oleifera* leaf powder with inert calcium sulphate powder, and observed that although calcium sulphate could reduce bacterial load on hands, the reduction was significantly smaller than obtained using 4 g of *Moringa oleifera* or non-medicated soap. These results suggest that *Moringa oleifera* has an extra effect due to its active components that cannot be attributed only to the mechanical action of rubbing hands together.

Many regulatory agencies require hand-wash (except for non-medicated soap) and hand-rub agents used in health care settings to meet certain performance standards when assessed using standardized test protocols [24,25]. In Europe, the most commonly used laboratory based *in vivo* methods to test antiseptics are those of the European Committee for Standardization (CEN) [26,27]. EN 1499 [14] and EN 1500 [28] belong to the category of CEN tests designed to evaluate the ability (of hand-wash or hand-rub agents, respectively) to eliminate transient pathogens from health care workers’ hands. This study followed an adaptation of protocol EN1499 which is designed to test the antimicrobial activity of hand wash agents. According to EN 1449, the mean reduction caused by the test product in the number of viable bacteria has to be significantly higher than that obtained with the reference soap [14]. 4 g of dried or wet *Moringa oleifera* did not pass this criterion, but it met the EN1500 requirement for a hand-rubbing agent, which said that the mean reduction in the numbers of viable bacteria shall not be significantly inferior to that with the reference product [28]. It can be argued that this product could also meet other standards used in different parts of the world. For example, in USA and Canada such formulations are regulated by the Food and Drug Administration (FDA) and Health Canada, respectively which refer to the standards of ASTM International [29]. Thus, a formulation may meet one criterion, but not another [30]. For example, in a study by Kramer et al. [30], various alcohol-based hand-rub gels accepted by ASTM standards failed to pass the European standards. At present, it remains unknown which requirement is the right one or what reduction in microbial release is needed to produce a meaningful reduction in the hand-borne spread of pathogens [31,32]. In this study we have demonstrated that 4 g of *Moringa oleifera* powder has the same effect as non-medicated soap, as both can significantly reduce bacteria counts in artificially contaminated hands. However we suggest this product is more suitable for domestic settings, rather than healthcare settings, where other products have already been demonstrated to be effective and available, and where the use of this powder would be less convenient.

The challenges of using this product in a domestic setting should be explored. How would users accept using a dried plant to clean their hands? How could the material be made available in specific domestic points where hand hygiene takes place? One possibility would be to place baskets with dried *Moringa oleifera* powder and spoons to dispense it, close to crucial places for hand hygiene, for example next to the cooking place and the latrine.

In this study we have demonstrated the effect of dried *Moringa oleifera* leaves in reducing bacteria on hands. It would be very interesting to test the bacterial reduction effect of fresh leaves. The possibility of using fresh leaves could facilitate its use as preparation process would not be necessary, opening up new avenues for its use, for example in open defecation places where Moringa trees are available.

## Conclusion

Four grams of *Moringa oleifera* powder in dried and wet application had the same effect as non-medicated soap when used for hand washing. *Moringa oleifera* could be very useful in places where soap or water is not available, and where this tree grows naturally. It could also be a cheap and healthy hand-washing optional product. Our data have been obtained in a laboratory setting, so the next step will be to try this product in real conditions and study its acceptability and convenience for potential users. *Moringa oleifera* formulations should be investigated further in randomized controlled trials.

## Competing interests

The authors declare that they have no competing interests.

## Authors’ contributions

Idea for the research question: SC, BB, BT. Design of the experiments: BT, DO. Performed the experiments: DO. Analyzed the data: BT, DO. Contributed reagents/materials/techniques: EC. Wrote the paper: BT. Comments and review of paper: SC, DO, EC. All authors’ read and approved the final manuscript.

## Pre-publication history

The pre-publication history for this paper can be accessed here:

http://www.biomedcentral.com/1472-6882/14/57/prepub
